# Ipsi- and Contralateral Moxibustion Generate Similar Analgesic Effect on Inflammatory Pain

**DOI:** 10.1155/2019/1807287

**Published:** 2019-02-05

**Authors:** Chuan-Yi Zuo, Peng Lv, Cheng-Shun Zhang, Ru-Xue Lei, Wei Zhou, Qiao-Feng Wu, Ling Luo, Yong Tang, Hai-Yan Yin, Shu-Guang Yu

**Affiliations:** Acupuncture and Tuina School, Chengdu University of Traditional Chinese Medicine, Chengdu 610075, Sichuan, China

## Abstract

The aim of this study was to investigate whether contralateral moxibustion would generate a similar analgesic effect with ipsilateral moxibustion. Contra- and ipsilateral moxibustion were separately applied to Zusanli (ST36) acupoints of inflammatory pain mice. The analgesic effect was evaluated, respectively, by licking/biting time (LBT) of formalin-induced inflammatory pain and thermal withdrawal latency (TWL) of complete Freund's adjuvant- (CFA-) induced inflammatory pain. For formalin-induced pain, compared with formalin group, the total LBT of ipsi- and contralateral moxibustion reduced in both phase I and phase II, but there was no significant difference between ipsi- and contralateral moxibustion. For CFA-induced inflammatory pain, compared with CFA group, TWL of ipsi- and contra-Moxi groups increased immediately after moxibustion intervention; however there was no obvious difference between ipsi- and contralateral moxibustion at any timepoint. It indicated that contralateral moxibustion had a similar analgesic effect with ipsilateral moxibustion in both formalin- and CFA-induced pain. These results suggest that both ipsi- and contralateral moxibustion could be applied for pain relief.

## 1. Introduction

Moxibustion, as an important member of traditional Chinese medicine (TCM) therapies, shares equal values with acupuncture in treating diseases in the traditional medical system of China [1]. It has been applied for more than 3000 years for a variety of diseases, especially for pain [2]. Different from the mechanical stimulation of acupuncture, the function of moxibustion mainly depends on heat stimulation, produced by igniting moxa sticks that are mostly made from mugwort (Artemisia vulgaris from traditional Chinese medicine). Moxibustion therapy is manipulated with igniting moxa stick at acupoints or some specific parts of the body [3]. Since most acupoints are symmetrically distributed on both sides of the body, here is a question: which side of acupoints could generate a better pain-relief effect? Previous studies demonstrated that contralateral acupuncture (contra-Acu, manual acupuncture on contralateral side) and contralateral electroacupuncture (contra-EA, electronic-acupuncture on contralateral side) could be applied in various diseases, including episodic cluster headache [2], chronic shoulder pain [4], hemiplegia following acute ischemic stroke [5], female lumbago and pain of the lower abdomen [6], spinogenic dizziness [7], and so on. These studies illustrated a similar effect of contra-Acu or contra-EA with ipsilateral acupuncture (ipsi-Acu, manual acupuncture on ipsilateral side) or ipsilateral electroacupuncture (ipsi-EA, electronic-acupuncture on ipsilateral side), while it still remains unclear that whether contralateral moxibustion (contra-Moxi, moxibustion on contralateral side) could also generate a similar effect in pain relief with ipsilateral moxibustion (ipsi-Moxi, moxibustion on ipsilateral side).

Inflammatory pain is a common clinical symptom of inflammatory diseases characterized by hyperalgesia, due to the sensitization of primary nociceptive neurons resulting from wounds, surgical incisions, burn injury, arthritis, infection, allergic reactions, autoimmune diseases, tumor growth, and other forms of tissue injury or disease [8]. Both complete Freund's adjuvant- (CFA-) and formalin-induced inflammatory pain models are commonly used to investigate inflammatory pain. CFA-induced inflammatory pain is a well-characterized model of chronic inflammatory pain [9]. The formalin-induced acute inflammatory pain could be divided into two phases: the first phase (phase I, 0–5 min) is caused by the initial tissue injury and a direct activation of peripheral small afferent fibers by formalin; the second phase (phase II, 15–60 min) is considered to be mediated by a low level of peripheral nerve activity, whose effect is then enhanced in the spinal level by central sensitization [10]. In this study, both formalin- and CFA-induced inflammatory pain models were established to compare the analgesic effect between contra-Moxi and ipsi-Moxi.

## 2. Materials and Methods

### 2.1. Animals

Adult male C57BL/6J mice, weighing 20-25g, were purchased from Beijing Hua Fu Kang Bioscience Co., Ltd. All mice were maintained on 12-h light/dark cycle, 24°C, and 40-50% humidity conditions, with free access to food and water. After being adaptively fed for one week, mice for formalin-induced inflammatory pain research were randomly assigned into control group, formalin group, ipsi-Moxi group, and contra-Moxi group, and mice for CFA-induced inflammatory pain research were randomly divided into control, CFA, ipsi-Moxi, contra-Moxi, saline + ipsi-Moxi, saline + contra-Moxi, ipsi-smoke-free Moxi, and moxa smoke groups. After all the experiments, mice were inhaled with isoflurane for anesthesia and then applied with cervical dislocation. All efforts were made to minimize suffering. All animal experiments were conducted in accordance with the National Institutes of Health (NIH) Guide for the Care and Use of Laboratory Animals [11] and the ethical guidelines of the International Association for the Study of Pain [12]. All experimental procedures were approved by the Animal Care and Use Committee of Chengdu University of Traditional Chinese Medicine (No. AECCDUTCM-2015-04).

### 2.2. Inflammatory Pain Model

The formalin-induced inflammatory pain models were prepared by injecting 10 *µ*l of 1 % (v/v) formalin solution (Sigma-Aldrich, St. Louis, MO, the concentration was adjusted using sterile saline) into the plantar surface of the left hind paw of mice [10]. Mice in the control group were injected 10*μ*l sterile saline (Kelun Industry Group, Sichuan, China) instead of formalin. The CFA-induced inflammatory pain models were established by injecting 20 *µ*l of 0.5 mg/ml complete Freund's adjuvant (CFA, Sigma-Aldrich, St. Louis, MO) into the plantar surface of left hind paws [9]. In the control group, mice were injected 20 *μ*l normal saline.

### 2.3. Moxibustion Intervention

**Figure 1:**
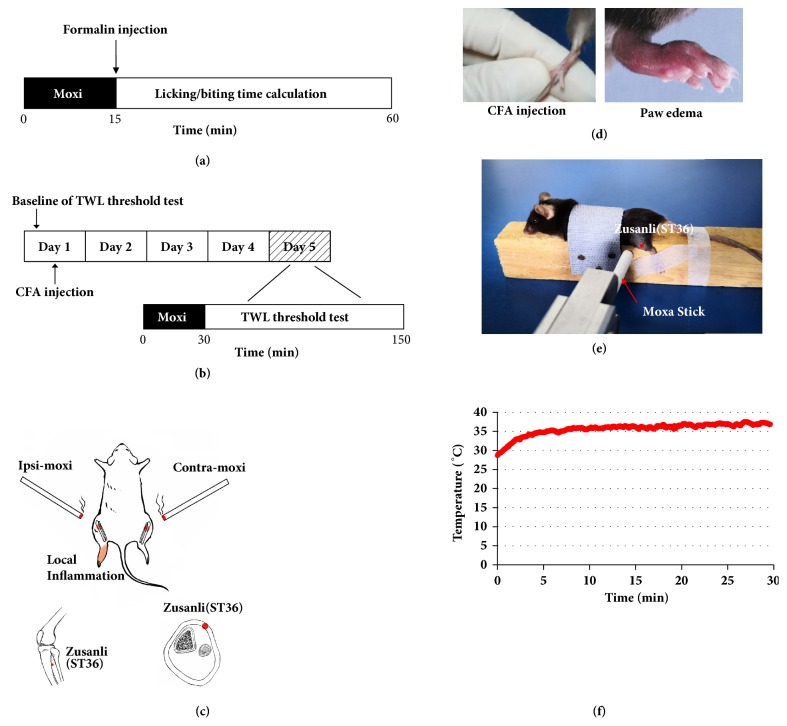
Schematic diagrams of experimental procedures and moxibustion intervention. (a) Experimental procedure on formalin-induced pain model: moxibustion intervention for 15min was given before formalin injection; mice received Moxi or restriction alone before formalin intraplantar injection. (b) Experimental procedure on CFA-induced pain model. On day 1, mice received CFA intraplantar injection after baseline of TWL threshold test. On day 5, mice were intervened by moxibustion for 30 min and then got TWL threshold test for 2h. (c) Mice received formalin/CFA injection in the left hind paw: ipsi/contra-Moxi was applied to ST36 in the left/right legs of mice respectively. (d) CFA injection and paw edema on day 5. (e) Moxibustion intervention. (f) Temperature curves on ST36: the lowest temperature: 28.7°C; the highest temperature: 37.5°C; temperature difference: 8.8°C.

Moxibustion was carried out respectively for 15 min on formalin-induced pain model before formalin injection (Figure 1(a)) and for 30 min on the fifth day on CFA-induced pain model after CFA injection (Figure 1(b)). Before moxibustion intervention, the fur on moxibustion site was shaved to expose ST36 (*Zusanli* acupoint, located 2 mm lateral to the anterior tubercle of the tibia in the anterior tibial muscle and 4mm distal to the knee joint lower point, Figure 1(c)). Ipsi-Moxi was done over the left ST36 while contra-Moxi was applied at the right ST36 (Figure 1(c)). For ipsi-smoke-free Moxi group, moxa smoke was eliminated by C200 smoke purifier (Shenzhen Conyson Company, Guangzhou, China). During moxibustion operation, the distance between the skin of ST36 and the lighted end of animal-used moxa sticks (length: 120 mm, diameter: 5 mm, Nanyang Hanyi Moxibustion Technology Development Co., Ltd., China, Figure 1(e)) was controlled within 1-1.5 cm. All procedures were performed at room temperature (24°C). The control and the model groups without moxibustion operation were under the same condition.

### 2.4. Pain Threshold Measurements

#### 2.4.1. Licking/Biting Times Calculation

As previously described [13], spontaneous behaviors following intraplantar formalin injection were measured. Mice were firstly acclimatized for 60 min in an individual Plexiglas cage placed atop a glass surface and then were videotaped following intradermal formalin injection and analyzed for nociceptive behaviors (defined as licking, lifting, and flinching of the injected paw, sum of licking/biting times were counted) in five min bins for 60 min following formalin injection. The first phase of spontaneous behavior was defined as 0–10 min after injection and the second phase of testing was defined as 10–45 min after injection. The procedure was done at room temperature (24°C).

#### 2.4.2. Thermal Threshold Measurement

As previously described [14], the thermal threshold was assessed by measuring the thermal withdrawal latency (TWL) with a Plantar Test Apparatus (Hargreaves method, PL-200, Tai Meng, China). Mice were placed in a transparent, square, and bottomless acrylic chamber (9 cm×8 cm×6 cm). After 15 min of habituation period, the infrared source placed under a glass plate was positioned near the operator directly beneath the plantar surface of the left hind paw. Withdrawal of the paw, indicating the sensation of pain in the mice, caused the infrared source to switch to the off position and the reaction time counter to stop. To avoid tissue damage, a resting interval of at least 5 min was set between tests, and the maximum time of heat focus was 20 sec. Each mouse was tested for three times, and the average value was set as the TWL. All experiments were performed at room temperature (approximately 24°C) and the stimuli were applied only when the animals were calm but not sleeping or grooming.

### 2.5. Statistical Analysis

Results are expressed as mean ± standard error of mean (SEM). Statistical evaluation was carried out by GraphPad Prism 6 (GraphPad Software, San Diego, CA, USA). Licking/biting time calculation results were evaluated by One-way Analysis of Variance (ANOVA) followed by Tukey-Kramer test. Thermal threshold measurement results were evaluated by repeated measures two-way ANOVA followed by Tukey-Kramer test.* P *values < 0.05 were considered statistically significant.

## 3. Results

### 3.1. Pain Threshold of Inflammatory Pain Mice

**Figure 2:**
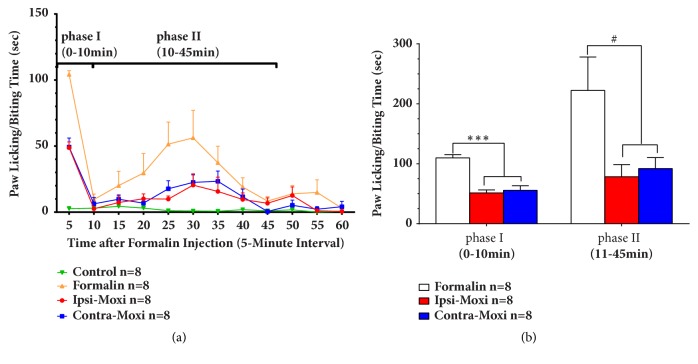
Analgesic effect of ipsi- and contralateral Moxi on formalin-induced pain model. (a) LBT of each timepoint from 0 min to 60min with 5 min interval: compared with formalin group, paw LBT decreased in control, ipsilateral, and contralateral groups (control: saline injection group; CFA: formalin injection group; ipsi-Moxi: ipsilateral moxibustion group; contra-Moxi: contralateral moxibustion group). (b) Total LBT of phase I and phase II: F (2, 21) = 32.53 (intervention between columns), contra/ipsi-Moxi versus formalin of phase I, *∗∗∗p *< 0.001; F (2, 21) = 4.945 (intervention between columns), contra/ipsi-Moxi versus formalin of phase II, #*p* < 0.05.

Pain threshold of formalin-induced inflammatory pain mice was detected from 0 min to 60 min with a 5 min interval following moxibustion for 15 min and then formalin injection. The processes of formalin test can be divided into two phases: an early phase lasting the first 5 min is due to direct effect on nociceptors and a late phase lasting from 20 to 30 min after the injection of formalin is considered as an inflammatory response with inflammatory pain. The LBT-pain threshold of the control group did not display any significant difference at different time point, the LBT of formalin group increased in general, and the LBT of contra- and ipsilateral Moxi groups decreased in general (Figure 2(a)). We calculated the total LBT of phase I and phase II; the total LBT of phase I and phase II of the formalin group significantly increased, while the total LBT of phase I and phase II of contra- and ipsilateral Moxi groups reduced, and there was no obvious difference between contra- and ipsilateral Moxi neither in phase I nor in phase II (Figure 2(b)). These data suggested that ipsi- and contralateral moxibustion had a similar effect on formalin-induced inflammatory pain.

### 3.2. Pain Threshold of CFA-Induced Inflammatory Pain Mice

**Figure 3:**
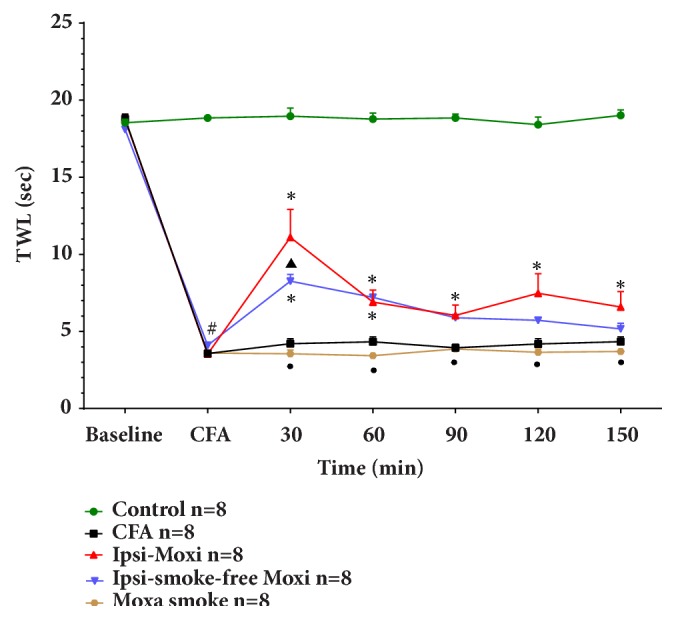
Analgesic effect of ipsi-Moxi, ipsi-smoke-free Moxi, and moxa smoke groups on CFA-induced pain model. In total, F (6, 210) = 418.4,* P*<0.0001 (time), F (4, 35) = 277.3,* P*<0.0001 (group), and F (24, 210) = 33.55,* P*<0.0001 (interaction). CFA versus control, #*P*<0.01; ipsi-Moxi versus CFA, ipsi nonsmoking Moxi versus CFA,*∗P*<0.05; ipsi-Moxi versus ipsi nonsmoking Moxi, ▲*P*<0.05; ipsi-Moxi versus moxa smoke, ●*P*<0.05 (control: saline injection group; CFA: CFA injection group, ipsi-Moxi: ipsilateral moxibustion group; ipsi-smoke-free Moxi: ipsilateral moxibustion with moxa smoke elimination group; moxa smoke: inhale moxa smoke group). There is no statistical difference between moxa smoke and CFA groups.

**Figure 4:**
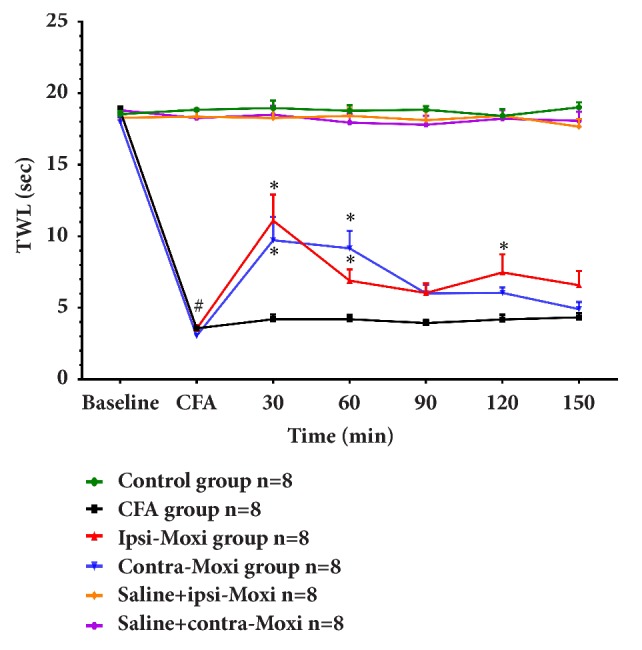
Analgesic effect of ipsi- and contralateral Moxi on normal and CFA-induced pain mice. In total, F (6, 252) = 114.2,* P*<0.0001 (time), F (5, 42) = 254.2,* P*<0.0001 (group), and F (30, 252) = 24.39,* P*<0.0001 (interaction). There is no statistical difference among control, saline + ipsi-Moxi, and saline + contra-Moxi groups (control: saline injection group, saline + ipsi-Moxi: saline injection with ipsilateral moxibustion group; saline + contra-Moxi: saline injection with contralateral moxibustion group). CFA versus control, #*P*<0.01; ipsi-Moxi versus CFA, contra-Moxi versus CFA, *∗P*<0.05 (control: saline injection group; CFA: CFA injection group; ipsi-Moxi: ipsilateral moxibustion group; contra-Moxi: contralateral moxibustion group). The difference between ipsi-Moxi and contra-Moxi groups has no statistical significance.

The pain threshold-thermal withdrawal latency (TWL) of CFA-induced inflammatory pain mice was detected on the fifth day and immediately followed with moxibustion intervention for 30 min. TWL baseline of each group displayed no significant difference, and after CFA injection, TWL of CFA and moxibustion groups decreased remarkably. There was no statistical difference between moxa smoke and CFA groups, but TWL of ipsi-Moxi and ipsi-smoke-free Moxi groups increased immediately after intervention, reached a peak after moxibustion for 30 min, and began to decline and closed to baseline after 2 h (Figure 3). On the whole, moxa smoke failed to generate analgesic effect and there was no big difference between ipsi-Moxi and ipsi-smoke-free Moxi groups, which indicated that the analgesic effect mainly produced by heat stimulation instead of moxa smoke. Ipsi- and contra-Moxi were also applied with saline injected mice. The result showed that there was no statistical difference among control, saline + ipsi-Moxi, and saline + contra-Moxi groups, which meant the analgesic effect induced mainly in inflammatory states (Figure 4), while, with CFA injection, TWL of ipsi- and contra-Moxi groups increased remarkably and reached a peak after moxibustion for 30 min, and then they began to decline and closed to baseline after 2h (Figure 4). These data suggested that both ipsi- and contralateral moxibustion generated a similar analgesic effect on CFA-induced inflammatory pain. However, ipsi- and contra-Moxi failed to induce analgesic effect in the mice without inflammatory pain syndrome.

## 4. Discussion

In this study, we mainly found that ipsi- and contralateral moxibustion achieved similar analgesic effect in both formalin-induced acute inflammatory pain and CFA-induced chronic inflammatory pain. In formalin-induced pain experiment, the similar analgesic effect of ipsi- and contralateral moxibustion could be found in phase I and phase II. In CFA-induced pain experiment, the similar analgesic effect of ipsi- and contralateral moxibustion could be lasted for 120 min. As we know, formalin-induced inflammatory pain includes two different phases: phase I is mainly due to direct chemical stimulation of peripheral nociceptors and phase II is generated by the ongoing stimulation of nociceptors by inflammatory mediators and/or by a first phase-induced spinal cord hyperexcitability, i.e., central sensitization [15, 16]. CFA-induced inflammation consists of a persistent unilateral inflammation generating hyperactivity of primary afferents from dorsal root ganglia and hyperexcitation of spinal dorsal horn interneurons [17]. Hence, our results implied that contralateral moxibustion alleviated pain symptom mainly through the central nervous system instead of local analgesia.

In this experiment, we also tested the local skin temperature of ST36 to exclude potential influencing factors, such as stress-induced analgesia. Then we found that the highest skin temperature of moxibustion site was 37.5°C, which was considered as a warm temperature and could not induce whole-body heat stress [18, 19]. Given that moxibustion was used for both preventing and treating pain symptom in clinic, ipsi- and contralateral moxibustion interventions were applied with saline injection, and it seemed that both ipsi- and contralateral moxibustion failed to induce pain tolerance of mice in physiological states. It also indicated that contralateral moxibustion, though giving moxibustion treatment at the unaffected side, could merely increase pain threshold of mice in pathological conditions. On the other hand, it is known that moxa smoke and thermal stimulation are produced simultaneously during moxibustion and there was research suggested that moxa smoke contributed to the therapeutic effect of moxibustion in some diseases, like wound infections, common warts, anal fistula, and so forth [20]. Since moxa smoke was produced in both ipsi- and contralateral moxibustion, moxa smoke and smoke-free moxibustion were applied separately, and the results showed that giving moxa smoke could not affect the pain threshold of mice. Therefore, these results confirmed that contralateral moxibustion had a similar analgesic effect with ipsilateral moxibustion.

Current data implied that applying moxibustion at any side of acupoint ST36 relieved inflammatory pain symptom and this found might be very useful for a more widespread application of moxibustion in different conditions. Contralateral moxibustion is able to be called* Jujiu* just like contralateral acupuncture, which is also named* Juci* or* Miuci* [21, 22], one of the traditional acupuncture techniques recorded in the classic textbook* Huang Di Nei Jing* [23]. For instance, some of the acupoints are located at the thin and tender skin places with many capillaries and these points are not allowed to be stimulated by moxibustion for a long time. Switching the side acupoints could reduce the potential damage to the local skin and capillaries, but still guarantee the treatment effect. Many classic TCM books also pointed out that it was not suitable to apply moxibustion at the acupoints with local skin lesion, such as local infection, trauma, anomaly, and postamputation, most of which were at the affected body side (ipsilateral side), for the reason that moxibustion at the affected side might increase the damage of local surface. In this case, it is more wisely to carry on moxibustion treatment at the alternative side, which had a similar effect with the affected side. However, the current study was only an animal experiment, and further clinical trials are needed to provide more powerful supporting data for the use of contralateral moxibustion.

Why contralateral and ipsilateral moxibustion could generate a similar effect? Our results indicated that the central nerve system might be crucial for analgesic effect of contralateral moxibustion. Since no similar study has been presented, it should be considered in our next study. On the other hand, underlying contralateral acupuncture and electroacupuncture (EA) have demonstrated that both peripheral and central nerve systems, including spinal interneurons [24, 25], endogenous opioids and diffuse noxious inhibitory controls (DNIC), were involved in the analgesic effects of acupuncture [26, 27]. These observations suggested that spinal interneurons were crucial in producing the contralateral acupuncture effect [28, 29]. It seemed that there were differences on the responses of subnucleus reticularis dorsalis (SRD) neurons between ipsilateral and contralateral acupuncture. The greater activation of total nociceptive convergence (TNC) than of partial nociceptive convergence (PNC) neurons was repeatedly observed when the ipsilateral part of the body was stimulated, and the responses of TNC neurons were slightly weaker from ipsilateral parts of the body than from their contralateral counterparts [24]. Another study showed that up-regulated expression of TRPV1 in dorsal root ganglia (DRG) and dorsal horn of the spinal cord (SCDH) after CFA injection was to be found on both ipsi- and contralateral EA stimulation [4]. The interactions between the two sides and projections of trigeminal afferents to the contralateral medullary horn also contribute to the contralateral stimulation [21, 30]. With functional magnetic resonance imaging (fMRI), researchers found that unilateral acupuncture stimulation was able to generate bilateral neuronal modulation [31, 32]. Additionally, a study indicated that the anterior cingulate cortex (ACC) could be one of the major regions for pain affection, which completely abolished analgesic effects of contra-EA, while the decrease of analgesic effects on ipsi-EA was still present [33]. Does contralateral moxibustion effect share the same mechanism with contralateral acupuncture or electroacupuncture? It is still far from clear. Therefore, it is quite necessary to clarify the mechanism of the antinociceptive effect induced by contralateral moxibustion in the future.

## 5. Conclusion

In conclusion, the present study indicated a similar analgesic effect between ipsi- and contralateral Moxi. These data supported the traditional acupuncture theory that stimulation could also be applied at the contralateral side in pain situation. However, to clarify the mechanism of the analgesic effect of Moxi, further dedicated investigation on this issue is required.

## Data Availability

The data used to support the findings of this study are available from the corresponding author upon request.
